# The quantitative impact of read mapping to non-native reference genomes in comparative RNA-Seq studies

**DOI:** 10.1371/journal.pone.0180904

**Published:** 2017-07-11

**Authors:** Adam Price, Cynthia Gibas

**Affiliations:** Department of Bioinformatics and Genomics, University of North Carolina at Charlotte, Charlotte, North Carolina, United States of America; Xiamen University, CHINA

## Abstract

Sequence read alignment to a reference genome is a fundamental step in many genomics studies. Accuracy in this fundamental step is crucial for correct interpretation of biological data. In cases where two or more closely related bacterial strains are being studied, a common approach is to simply map reads from all strains to a common reference genome, whether because there is no closed reference for some strains or for ease of comparison. The assumption is that the differences between bacterial strains are insignificant enough that the results of differential expression analysis will not be influenced by choice of reference. Genes that are common among the strains under study are used for differential expression analysis, while the remaining genes, which may fail to express in one sample or the other because they are simply absent, are analyzed separately. In this study, we investigate the practice of using a common reference in transcriptomic analysis. We analyze two multi-strain transcriptomic data sets that were initially presented in the literature as comparisons based on a common reference, but which have available closed genomic sequence for all strains, allowing a detailed examination of the impact of reference choice. We provide a method for identifying regions that are most affected by non-native alignments, leading to false positives in differential expression analysis, and perform an in depth analysis identifying the extent of expression loss. We also simulate several data sets to identify best practices for non-native reference use.

## Introduction

Sequence read alignment to a reference genome is currently a key step in many common bioinformatics workflows. Researchers frequently encounter situations in which the most appropriate reference genome for a reference-based analysis is not available, and a homologous alternative must be used. This can lead to inaccuracies in mapping and subsequently in quantitation and interpretation. These inaccuracies skew the results of otherwise sound analysis workflows. This study approaches the problem of non-native reference alignment by comparing the effects of read alignment to native and heterologous reference genomes. We describe a method to identify false positives caused by improper alignments to the heterologous reference, and examine the underlying causes, to provide a set of best practices for research that makes use of non-native reference genomes.

Comparative analysis of microbial genomes since the advent of high-throughput sequencing has shown that prokaryotic genomes are dynamic and can be highly diverse, even among closely related species or strains. Analysis of bacterial genomes through sequencing-based methods such as RNA-Seq has made it possible to rapidly advance our understanding of basic biological function, identify host-pathogen interactions, and engineer microbes for industrial and pharmaceutical applications [1]. It has become apparent in recent years that the highly dynamic nature of prokaryotes has led to extensive genomic diversity. In 2001, sequencing of *E*. *coli* O157:H7 identified over 1300 strain-specific genes when compared with *E*. *coli* K-12, the strain previously sequenced and thought at the time to be fairly representative of the model organism [2]. The identification of these genes, found to be involved primarily in virulence and metabolism, showed that even closely related strains can differ significantly. Since that time, the availability of sequencing data from multiple strains of the same organism has increased, but due to the vastness of biodiversity in prokaryotes, it is still not uncommon to find that the most appropriate reference genome is not available, or exists only as a draft. Researchers then must resort to using finished evolutionary neighbor reference genomes in their studies, even when the sequence reads they wish to map were produced from a heterologous strain.

Many common analysis pipelines rely on accurate alignment of reads to a corresponding reference genome. Differential expression studies, for example, rely on aligning transcriptome reads to a reference, extracting count data, and examining the differences in transcript read levels for the genome under study. In cases where two or more closely related species or strains are being studied, a common approach is to simply map reads from all organisms to a common reference genome. The assumption is that the differences between closely related microbes are insignificant enough that the results of differential expression will not be influenced, or otherwise, that genes that are absent in one sample or the other should simply be excluded from analysis, while shared genes that have seemingly reasonable read counts in both organisms can be used for differential expression analysis. For example, we previously investigated differences in gene expression in clinical strains of *Vibrio vulnificus*, when exposed to either artificial seawater or human serum environments, as a model for the expression changes the organism undergoes when infecting a human host. *V*. *vulnificus* CMCP6 and *V*. *vulnificus* YJ016 expression levels were compared by using the CMCP6 strain as a common reference genome [3]. Using a common reference genome to make comparisons between different strains is also common in eukaryotic systems, and similar methods were used in a comparative study of strains of *Bombyx mori* [4]. The approach of using a common reference genome for different strains is unable to correctly represent factors that can influence read counts in the non-homologous read set, such as the frequency and density of mismatches due to natural divergence between strains. The degree of error in these studies will be affected by how alignment algorithms handle reads with multiple possible mapping positions, especially when mutations decrease mapping position certainty. Comparing data across strains becomes increasingly less sound as evolutionary distance between read sets and the reference genome increases, and this is particularly true of prokaryotic species, where divergence occurs at an accelerated pace. In this study, we examine the potential impact of using a heterologous reference genome and the effects on read alignment, and by extension differential expression. We show how differences in reference genomes influence read alignment and gene expression results when using common analysis techniques. We then provide an approach for identifying false positives caused when comparing multiple strains or species by means of alignment to a common reference genome, and outline best practices for the use of heterologous reference genomes in cross-strain analyses.

## Materials and methods

### Data and heterologous reference distance

For this study, transcriptome data from two different organisms were used. RNA-Seq data for two strains of *Vibrio vulnificus*, CMCP6 and YJ016, as described by Williams et al., were used. These data are available under the NCBI Bioproject identifier PRJNA252365. Another publicly available data set, consisting of RNA-Seq data from *Escherichia coli* strains K12 (MG1655), a common laboratory strain, and strain IAI1, a commensal modal strain, under three experimental conditions [5] was also analyzed (Bioproject identifier: PRJNA310115). The *V*. *vulnificus* data set consists of two experimental conditions, human serum and artificial seawater, each having two replicates, while the *E*. *coli* data set consists of three experimental conditions, batch, chemostat, and starvation, with each condition having two replicates as well. A summary of the data used in this study can be seen in Table 1. In all cases sequencing was performed using the Illumina HiSeq platform. The same analysis workflow was applied to each data set.

**Table 1:** Summary of read data, including average coverage when reads are mapped to native or heterologous genomes.

Species	Strain	Condition	Replicates	Coverage (Native)	Coverage (Heterologous)
*V*. *vulnificus*	CMCP6	Human Serum (HS)	2	628.5 / 671.2	678.9 / 522.9
*V*. *vulnificus*	CMCP6	Artificial Saltwater (ASW)	2	641.5 / 550.5	700.9 / 349.7
*V*. *vulnificus*	YJ016	Human Serum (HS)	2	715.8 / 544.4	595.3 / 611.8
*V*. *vulnificus*	YJ016	Artificial Saltwater (ASW)	2	709.3 / 350.1	616.1 / 531.2
*E*. *coli*	K12 (MG1655)	Batch	2	169.2 / 206.6	175.7 / 214.6
*E*. *coli*	K12 (MG1655)	Chemostat	2	194.4 / 136.8	201.9 / 142.4
*E*. *coli*	K12 (MG1655)	Starvation	2	108.6 / 107.4	115.3 / 113.9
*E*. *coli*	IAI1	Batch	2	145.9 / 121.9	154.4 / 128.4
*E*. *coli*	IAI1	Chemostat	2	135.1 / 130.9	142.9 / 138.5
*E*. *coli*	IAI1	Starvation	2	98.9 / 95.5	104.4 / 101.1

Structural comparisons between reference genomes for both bacterial strains were performed using Mauve [6].

### Orthology mapping

In order to make accurate comparisons of data as aligned to heterologous reference genomes, orthology relationships between genes were first determined. All-against-all protein BLAST was used to find orthologous regions between strains. Regions were determined to be orthologous if they showed greater than 95% identity, were at least 200 base pairs in length, and had no more than 5 mismatches at the protein level.

### RNA-Seq read mapping

**Fig 1:**
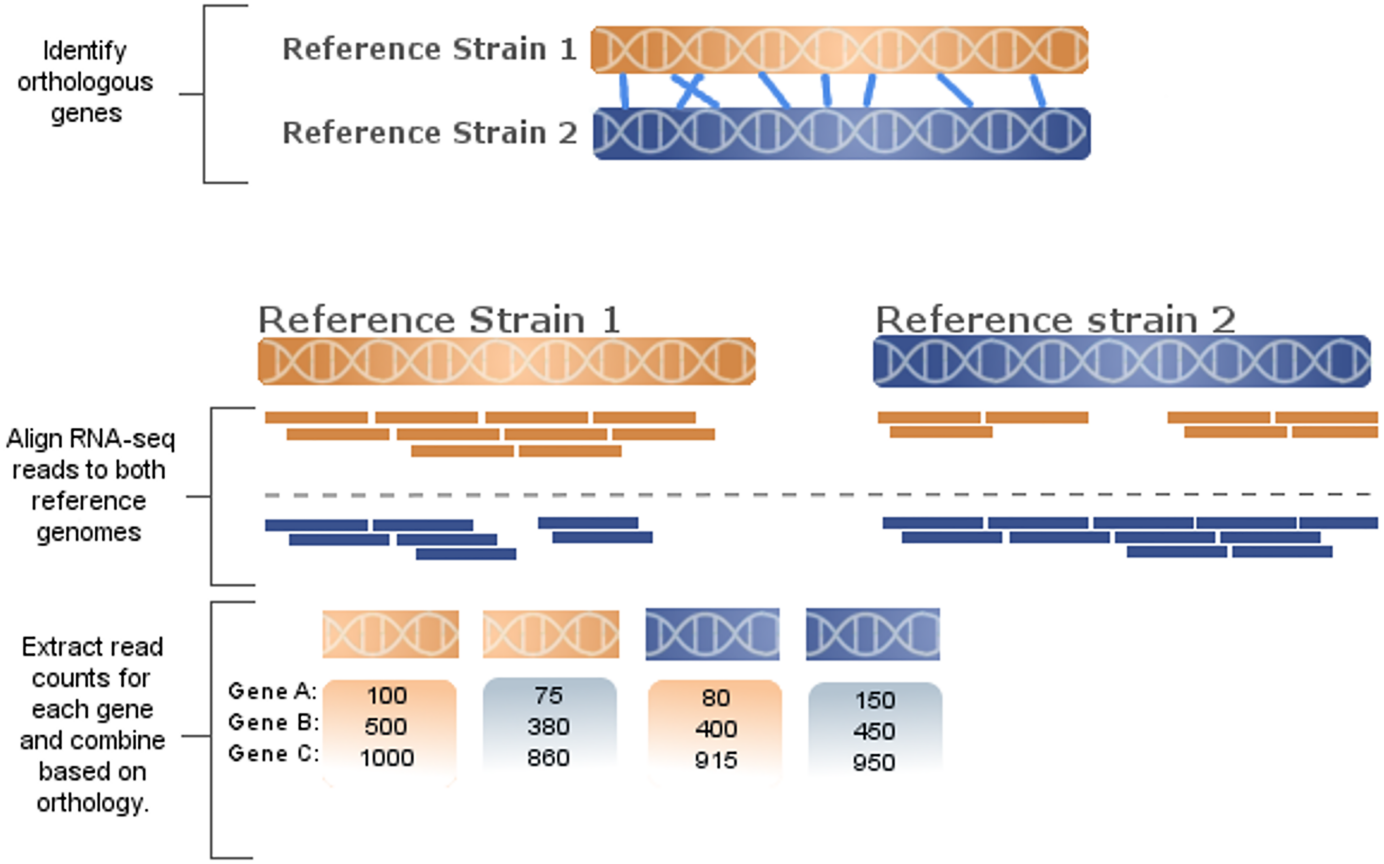
Data processing pipeline. Orthology is identified between heterologous strains and reads are aligned to both reference genomes. Using the orthology mapping information, extrapolated read alignment counts are compiled such that counts can be compared for each read set as aligned to each reference genome.

Each RNA-Seq read set was aligned to both potential reference genomes for their respective species using Bowtie2 [7]. In the case of *E*. *coli*, all replicates and conditions from both strains K12 and IAI1 were aligned to both the K12 and IAI1 reference genomes. Similarly, all read sets for *V*. *vulnificus* were aligned to both the CMCP6 and YJ016 reference genomes. All alignments were performed using Bowtie2’s sensitive alignment (-M 3, -N 0, -L 22). Raw read counts were then extracted from each alignment using the featureCounts Bioconductor package [8]. Next, the previously computed orthologous gene mapping information was used to map read count data for all conditions and replicates with orthologous genes. This process was performed on all samples and replicates for both *E*. *coli* and *V*. *vulnificus*, so that each read set is counted for alignment to both their native reference genome and the heterologous reference genome for their respective species. This makes it possible to make direct comparisons of the effects of mapping identical read data to heterologous genomes. As the process was applied for all conditions to both native and heterologous alignments, cross effects can be identified to increase confidence in observations. An overview of this data processing pipeline is shown in Fig 1.

### Differential expression analysis

Differential expression analysis was performed for all strain/condition permutations for each organism, using DESeq2 [9]. Principal component analysis was performed to confirm the integrity of replicates for all read sets.

### False positive identification

Differential expression analysis was performed on all permutations of data sets for each organism as aligned to native and heterologous reference genomes. False positives were identified in two ways. When identical datasets were aligned to both references for a single condition, differential expression analysis was performed and the set of differentially expressed genes were taken as false positives. For example, *E*. *coli* strain K12, batch condition was aligned to both native and heterologous genomes and differential expression was performed, identifying 15 false positives. When identifying false positives across multiple conditions, differential expression for two conditions is performed with both conditions aligned to native and heterologous reference genomes, and false positives are then identified as the set difference between the two differential gene results. For example, when comparing *E*. *coli* strain K12, batch condition to the K12 chemostat condition, differential expression is performed on the batch vs chemostat reads as aligned to the K12 genome, and then as aligned to the IAI1 genome. True positives are considered to be the intersection of these two result sets, and false positives are considered to be the difference of the two sets.

### Simulation of reads and genomes

For the simulations described, reference genomes were simulated using Simulome [10]. Reference genome simulations were based on the *E*. *coli* K12 strain. The simulated reference genome contained 500 genes, with lengths selected in a normal distribution around the mean length of genes for the K12 strain. Each simulated gene was separated from its neighbor by a randomly sized intergenic region. A heterologous version of this reference genome was also simulated, in which each gene contained 35 SNPs, approximately the average number of SNPs observed for false positives identified for *E*. *coli* that were caused by SNP induced read loss. Read data for the simulated genome was then simulated using the ART package [11]. Read data was created for the simulated reference genome based on ART’s Illumina HiSeq 2500 model, with simulated fold coverage of 150, for read lengths of 50,100, and 150. These parameters were selected to mirror the properties of the actual data for the *E*. *coli* data set. The simulated reads were aligned to the simulated reference, which was considered the “native” reference genome, and also to the simulated heterologous reference, in which each gene contained 35 randomly distributed SNPs across the length of each gene. Alignment and read count extraction methods were performed identically to those outlined in the methods section on the actual read data.

## Results

### Structural comparison of genomes

The *E*. *coli* K12 strain is 4,641,652 base pairs in length and the IAI1 strain is 4,700,560 long, for a difference in length of 58,908 base pairs. 64,577 SNPs were identified across the span of both strains, making the approximate level of polymorphic sites 1.38%. The combination of indel and polymorphic differences between these genomes is 2.64%. Structural analysis shows relatively few rearrangement events and broad similarity between these genomes.

**Fig 2:**
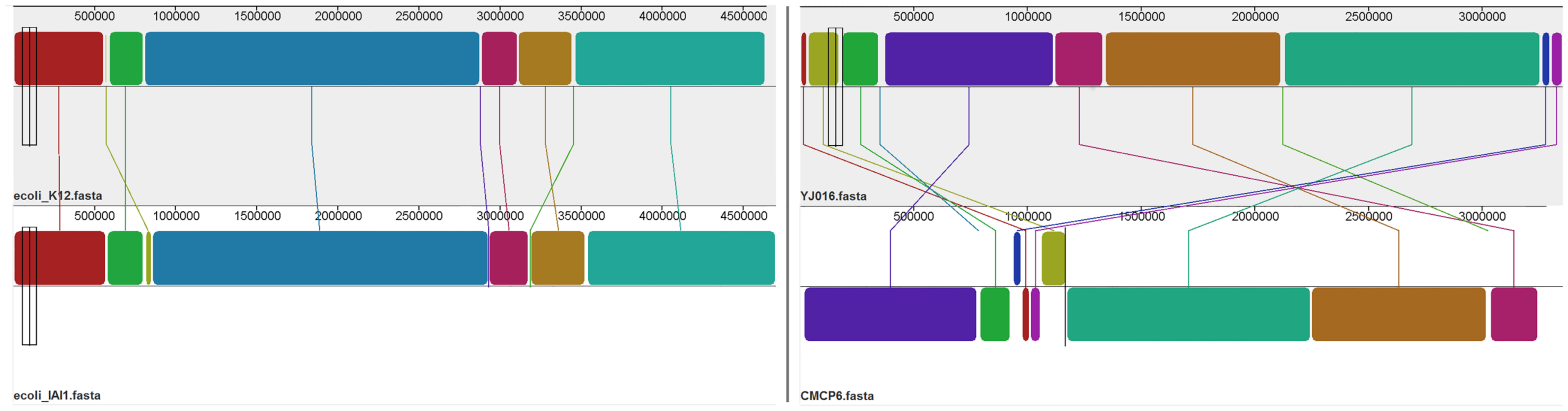
Structural differences between reference strains for *E*. *coli* (left) and V. Vulnificus (right). A 2.64% difference is observed between references in *E*. *coli* and a 3.61% difference is observed in *V*. *Vulnificus*, accounting for indels and polymorphic sites.

*V*. *vulnificus* CMCP6 and YJ016 are 3,281,866 and 3,354,505 base pairs in length respectively, for a difference in total length of 72,639 base pairs. 46,955 SNPs were identified between the two references, making the difference between the two genomes by polymorphic sites 1.42%. Combining the total differences for polymorphisms and indel events, these strains are approximately 3.61% different from one another. Structural analysis of the *V*. *vulnificus* genomes revealed more structural changes than were observed in *E*. *coli*, although overall structural similarity is still high. Fig 2 shows structural differences for both organisms.

1570 orthologous regions, approximately 36% of annotated genes, were identified between *V*. *vulnificus* strains CMCP6 and YJ016. 2378 orthologous regions, approximately 55% of genes, were identified between *E*. *coli* strains K12 and IAI1. Annotation information for each strain was then applied to these orthologous regions, to determine correspondence in read counts between strains at a per-gene level.

### False positive identification

By examining the results of differential expression analysis on identical read data, with the choice of reference genome being the only differential factor, any genes that are identified as being differentially expressed for the same condition can be marked as false positives caused by reference-based factors.

For example, by aligning reads from *E*. *coli* strain K12, batch condition, to both the K12 reference genome and the heterologous IAI1 reference genome, and then performing differential expression analysis, any genes that are identified as being differentially expressed can be assumed to have been incorrectly identified, as the initial read set is identical and the only differential factor is the reference genome. When examining the reciprocal condition, in which reads generated from the IAI1 strain, batch condition, are aligned to both reference genomes, another set of false positives can be identified, many of which correspond to the false positives identified previously, creating a cross-identification effect for many false positives.

**Fig 3:**
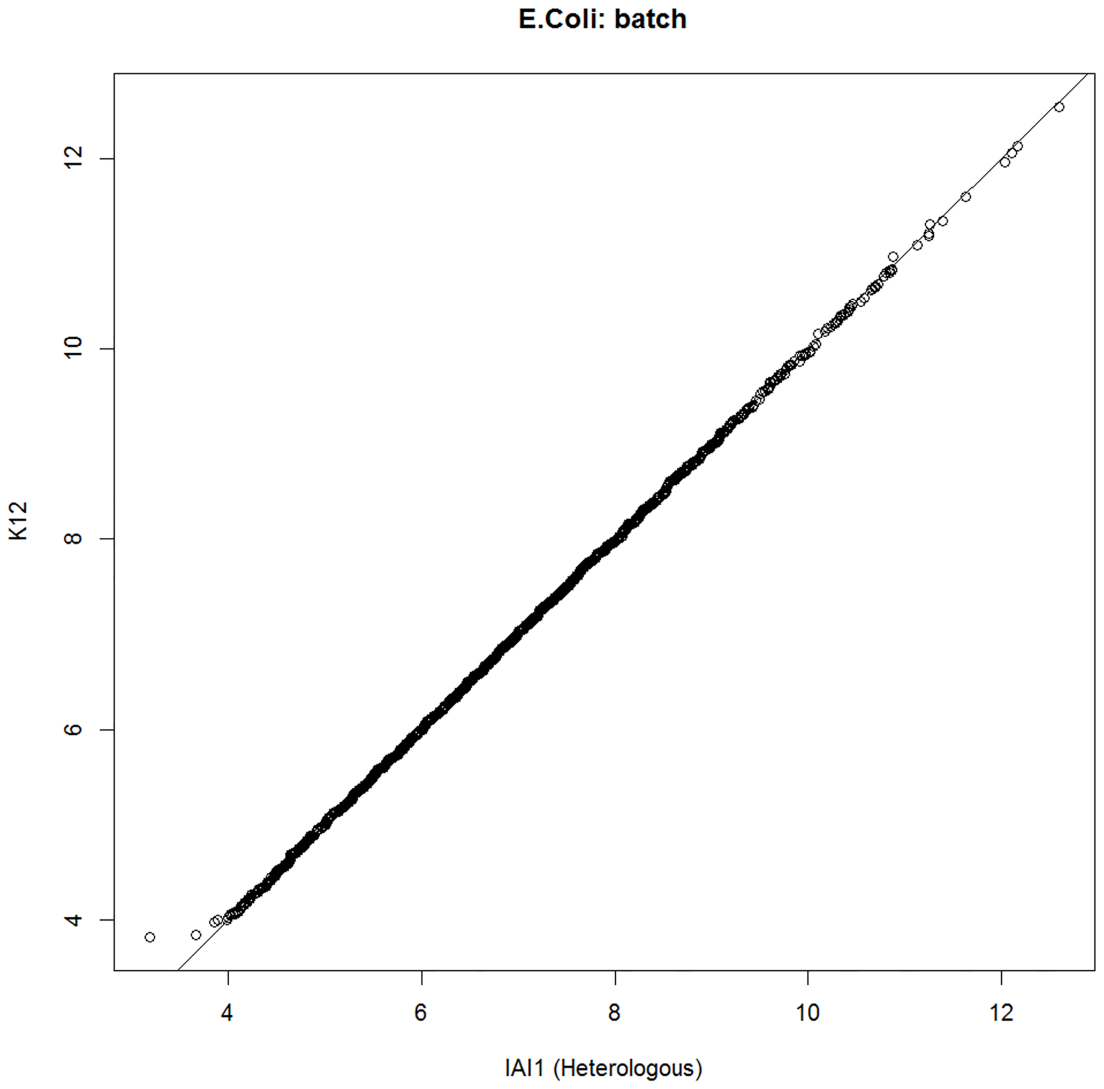
Log-fold changes of read counts for all *E*. *coli* strain K12 genes as aligned to both native and heterologous references. The same read sets as aligned to both reference genomes reveals the extent of bias present due to differences in the choice of reference genome.

Fig 3 shows an example of the log-fold changes for all genes for the *E*. *coli*, strain K12, batch condition. In this case, reads generated from strain K12, batch condition, were aligned to both the native reference and the heterologous reference, strain IAI1. As the reads are identical, high correspondence is naturally expected, with variation only being caused due to differences in the reference genome. High correspondence such as this was observed for all conditions and read sets. This level of correspondence indicates that the assumption that these two genomes can be used interchangeably as a reference for read alignment is reasonable. Had this data shown significant deviation, it would have indicated that heterologous alignment was not appropriate. This comparison of alignment to orthologous regions should be applied in cases when a reference genome for a read set is unavailable, but a homologous alternative exists, in order to determine if alignment to the homologous reference if viable. We will return to this point in more detail in the discussion section.

This method generally identifies significantly more false positives than are identified when only a single condition is examined. This compounding of false positives is to be expected as the first method relies on aligning only one read set to two references (2 replicates x 2 alignments each), and the second method must align two read sets to two references (2 replicates x 4 alignments each). Table 2 shows a summary of all false positives identified through both methods.

**Table 2:** Summary of differential expression false positives detected in non-native mapping cases.

Species	Native Reference	Condition	False Positives	Cross-identified
*V*. *vulnificus*	CMCP6	Human Serum (HS)	0	0
*V*. *vulnificus*	CMCP6	Artificial Saltwater (ASW)	2	1
*V*. *vulnificus*	YJ016	Human Serum (HS)	1	0
*V*. *vulnificus*	YJ016	Artificial Saltwater (ASW)	2	1
*V*. *vulnificus*	CMCP6	HS vs ASW	14	1
*V*. *vulnificus*	YJ016	HS vs ASW	14	1
*E*. *coli*	K12 (MG1655)	Batch	15	5
*E*. *coli*	K12 (MG1655)	Chemostat	6	4
*E*. *coli*	K12 (MG1655)	Starvation	1	1
*E*. *coli*	IAI1	Batch	9	5
*E*. *coli*	IAI1	Chemostat	16	4
*E*. *coli*	IAI1	Starvation	5	1
*E*. *coli*	K12 (MG1655)	Batch vs Chemostat	58	6
*E*. *coli*	K12 (MG1655)	Batch vs Starvation	17	0
*E*. *coli*	K12 (MG1655)	Chemostat vs Starvation	32	2
*E*. *coli*	IAI1	Batch vs Chemostat	61	6
*E*. *coli*	IAI1	Batch vs Starvation	40	0
*E*. *coli*	IAI1	Chemostat vs Starvation	42	2

Cross identification of false positives was also examined to determine if the same regions produce false positives across different read sets. Several false positives were identified from multiple read sets; however, cross identification is not necessarily always present due to naturally occurring differences in expression levels between different strains. Even though a reduction in read counts is typically associated with alignment to a heterologous genome, genes will not necessarily be identified as differentially expressed unless the log-fold change is significantly different with regard to the expected concentration of fragments as determined by dispersion of counts across the entire read set (9). For example, if an *E*. *coli* read set from the K12 strain is aligned to both a native and a heterologous genome, and a gene is identified as a false positive through differential expression, we can be confident that read alignment for that gene is being compromised by the reference genome. While it is likely that the same gene will be reciprocally compromised in the corresponding read set from the IAI1 strain, it may or may not be identified as differentially expressed because the overall expression levels in IAI1 may be naturally different from K12, and the log-fold change may not be extreme enough to identify the gene as differentially expressed with regard to fragment dispersion for the entire IAI1 read set. For this reason, genes are considered to be false positives if they are identified in either case, though special attention was given to genes with cross identification as representative cases of false positive causes in later analysis.

Read counts for false positives tended to be significantly higher when aligned to their native genome than their heterologous counterpart. This is to be expected, as it is likely that differences in genome cause alignment failures for non-native reads. Once false positives were identified, sequence analysis was performed. Nucleotide BLAST was performed on all ortholog pairs to examine the influence of reference sequences on read alignment. For *E*. *coli* the mean number of polymorphic sites per gene was 13 between the two reference genomes. Similarly, *V*. *vulnificus* strains showed a mean of 12 SNPs per ortholog pair. In all cases, false positives contained two to three-fold increases in SNPs, with *E*. *coli* having an average of 28 SNPs per false positive and *V*. *vulnificus* having 26. This ratio was also observed with regard to gene length, with the number of SNPs to length ratio for false positives being around three times that of true positives.

Once false positives were identified through differential expression analysis, further examination of the identified genes was conducted to identify the underlying causes for false positive identification. Two specific causes are identified as the primary contributing factors: indel/duplication events and high-density SNP windows.

### Indel & duplication events

**Fig 4:**
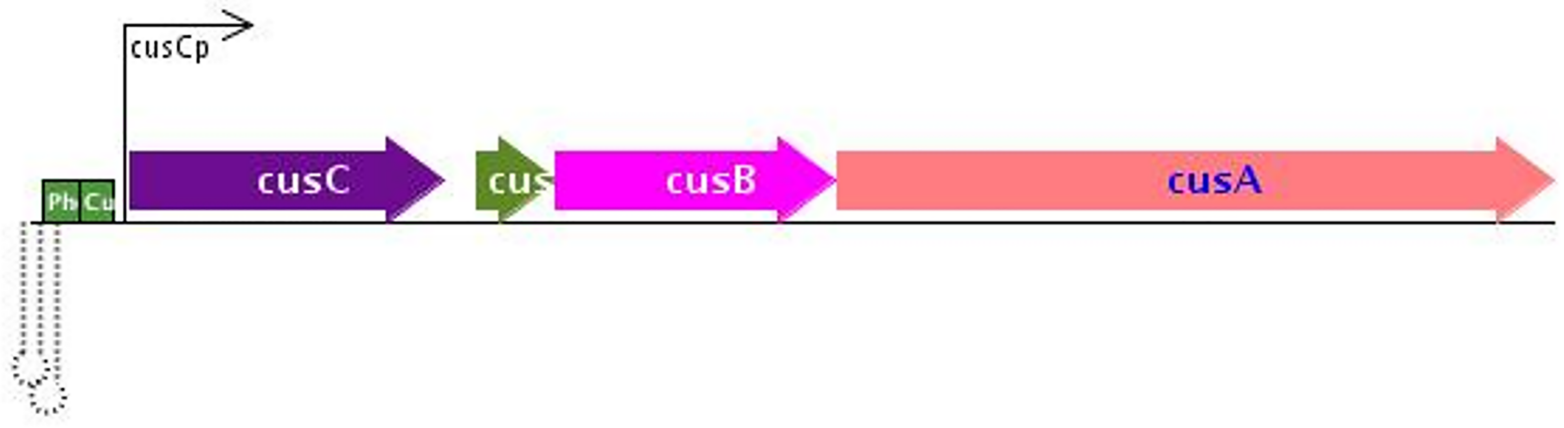
Operon structure containing the gene, cusC, which is responsible for copper resistance in *E*. *coli*. 21 SNPs are present across 1373 bases between the K12 and IAI1 reference strains.

One gene that was identified as a false positive of particular interest in *E*. *coli* was cusC. This gene was cross-identified for both batch and chemostat conditions for read sets generated from both the K12 and IAI1 strains. For this reason, this gene was selected as a representative sample for the explanation of duplication based false positive identification. cusC is the first gene of an operon consisting of 4 genes. Fig 4 shows an overview of the operon structure [12].

The cusC operon encodes a two-component signal transduction system that is responsive to copper ions, acting as a regulatory system to the pco operon, which provides copper resistance for *E*. *coli* [13]. The cusC gene itself is 1373 bases long and has 21 SNPs along its length between the K12 and IAI1 strains. Overall expression for this gene is generally low relative to average expression levels for each genome, and the ratio of SNPs to gene length is also approximately half that of typically identified false positives. Other genes in the operon following cusC are not identified as false positives.

**Fig 5:**
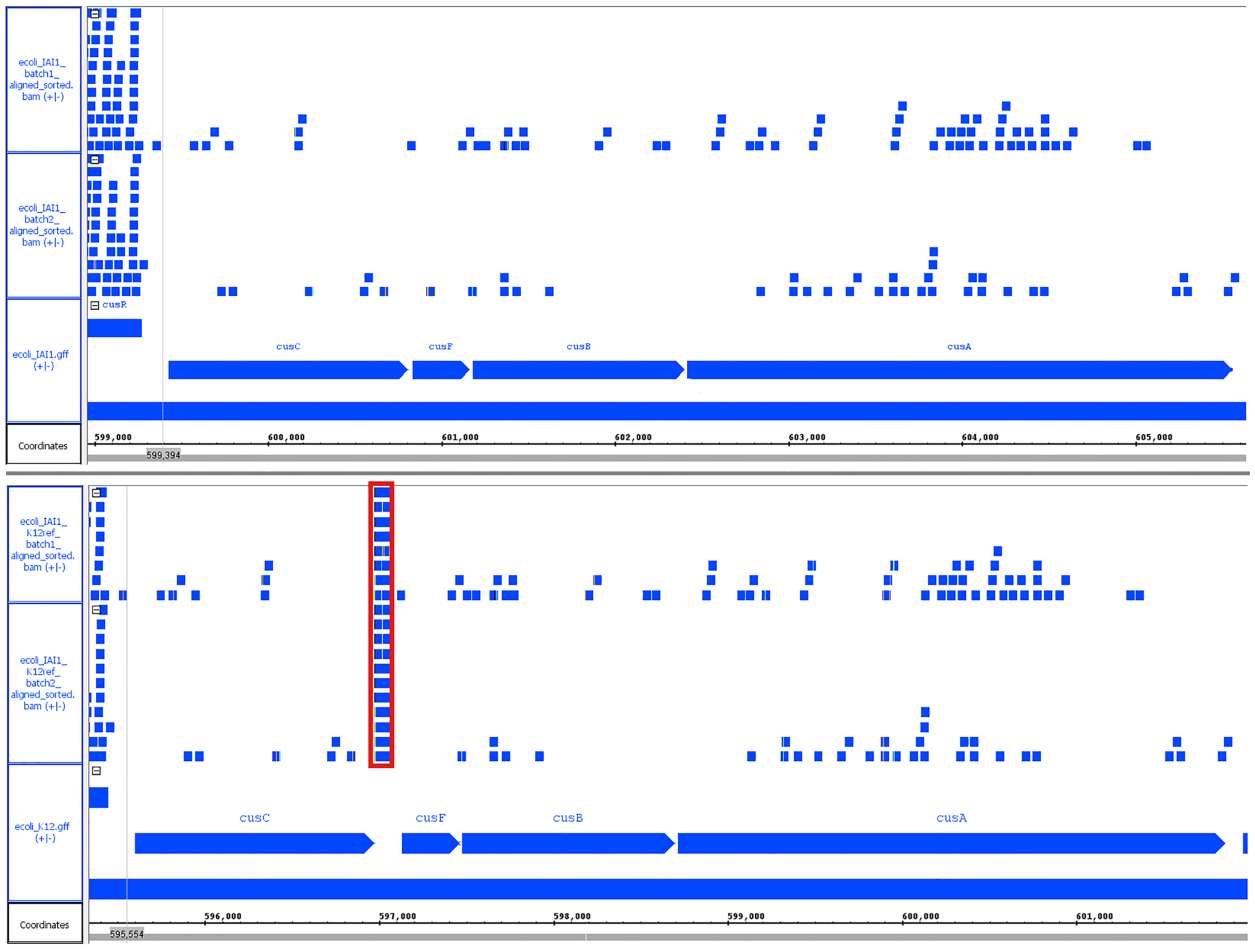
Read alignment for cusC operon for IAI1 batch condition, replicates 1 and 2. The native IAI1 genome (top) shows a continuous operon with sparsely aligned reads, while the heterologous K12 genome (bottom) shows an insertion after the cusC gene that is highly expressed (outlined in red).

Investigation into the cause of false positive identification of cusC found that incorrect expression levels are created due to an indel event between the two genomes. Fig 5 shows an example of read alignment to this operon for an identical read set as aligned to both native and heterologous genomes [14].

**Fig 6:**
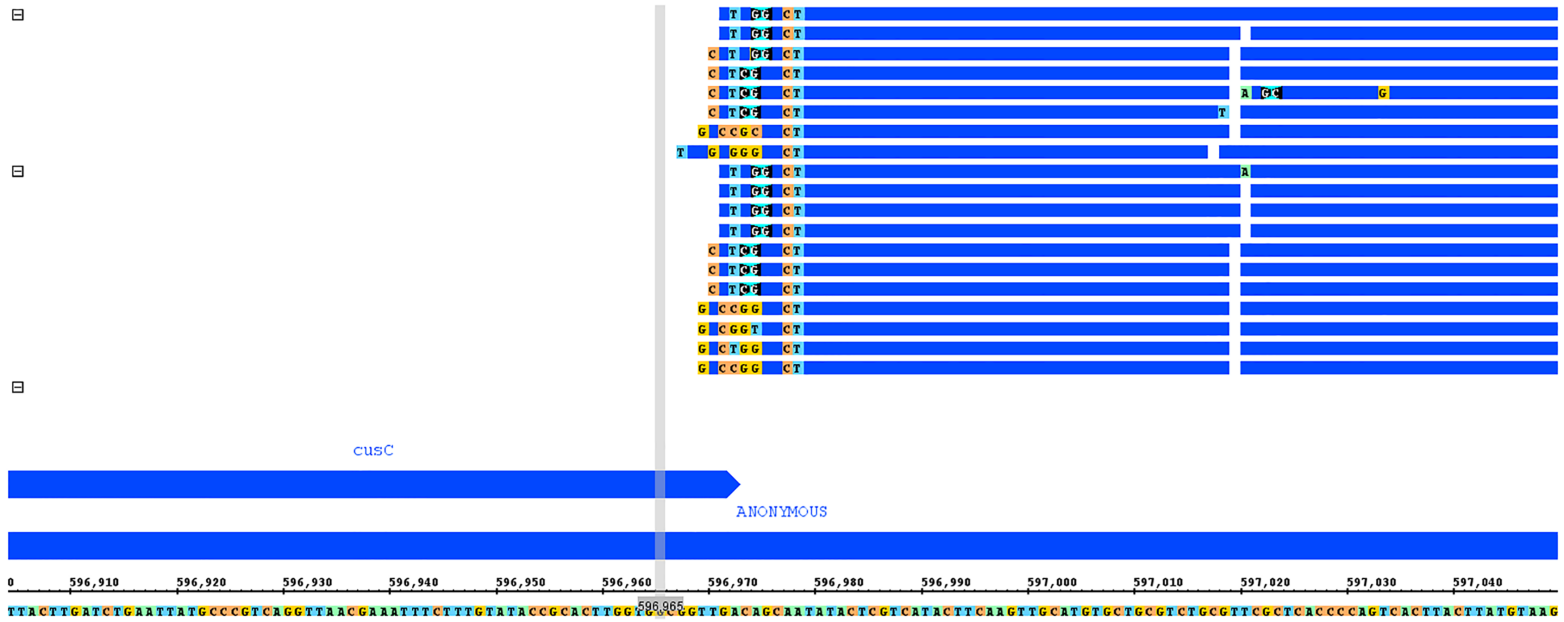
Reads in the indel region slightly overlapping cusC, with particularly poor alignment in the overlapping area. The overlapping region substantially biases expression counts for this gene in the non-native reference genome.

The indel event that can be seen between cusC and cusF between the native (IAI1) and heterologous (K12) genomes causes reads that align to the gapped area that slightly overlap cusC to be counted as expression for the cusC gene, causing a log-fold change in expression between the true and false expression levels of approximately 3.4, a highly significant difference. Examination of the overlapping region, as shown in Fig 6, shows that the reads map poorly to this region, especially in the area overlapping the cusC gene. In addition, the reads in this example were generated from the IAI1 strain and therefore cannot have produced reads in these positions, which implies that these genes are most likely the result of a duplication event and have been mapped to multiple locations. This suggests that elsewhere in the genome a region where these reads map accurately should exist. To investigate this possibility, BLAST was performed on the sequence from the indel point and found five matching positions in the K12 genome and only four in the IAI1 genome.

Each of these positions were individually inspected in both genomes and appear to be orthologous across references, with the additional matching region in the K12 strain being the observed location in cusC. In both genomes, a single case was found where these reads map perfectly, with all other cases showing similarly poor alignments to that shown in Fig 6. It is likely that a duplication occurred in *E*. *coli*, in which sequence from this section of genome, showing perfect alignment for these reads, was inserted into other parts of the genome. This specific sequence duplication has occurred in K12 in the cusC operon, but did not occur in the IAI1 strain. When alignment is performed using either the IAI1 or the K12 based reads, because the original sequence that was duplicated still exists elsewhere in both genomes and is expressed, reads from that region incorrectly map to the duplicated region in one genome and not the other, causing a false positive. Interestingly, one of the positions identified as a duplicated region for this same sequence corresponded to another gene, yhbI, which was cross identified as a false positive in all *E*. *coli* conditions and read sets. This gene shows the exact same expression profile, with a highly-expressed region of poorly mapped reads aligning near the end of the gene.

This type of improper alignment is due to how bowtie2 handles reads that map to multiple locations. When multiple sites are identified for possible alignment by bowtie2, reads can be mapped to both positions. For this reason, false positives are identified at points where small duplications have occurred within the genome and minimal divergence has occurred at the duplicated points. One possible solution to this might be to consider only uniquely mapped reads, however this would have the effect of removing all reads that map to multiple locations from all possible mapping positions, which would bias the data for the actual mapping position from the opposite direction, removing the false positive identification for cusC and yhbI, but changing the expression levels of the gene where the reads were truly expressed. This is discussed in further detail later in this study.

### SNPs

The other primary cause of false positive identification between native and heterologous genomes was read loss caused by SNPs in highly concentrated windows. A majority of false positives identified for all conditions showed significantly higher proportions of polymorphic sites for false positives on average as compared to the mean level of polymorphic sites between genes for the genomes as a whole. False positives identified due to read alignment loss due to SNPs showed a two to three-fold increase in propensity of SNPs with regard to their length, while genes identified as being false positives due to indel/duplication events showed sequence correspondence more similar to average expected levels of difference.

**Fig 7:**
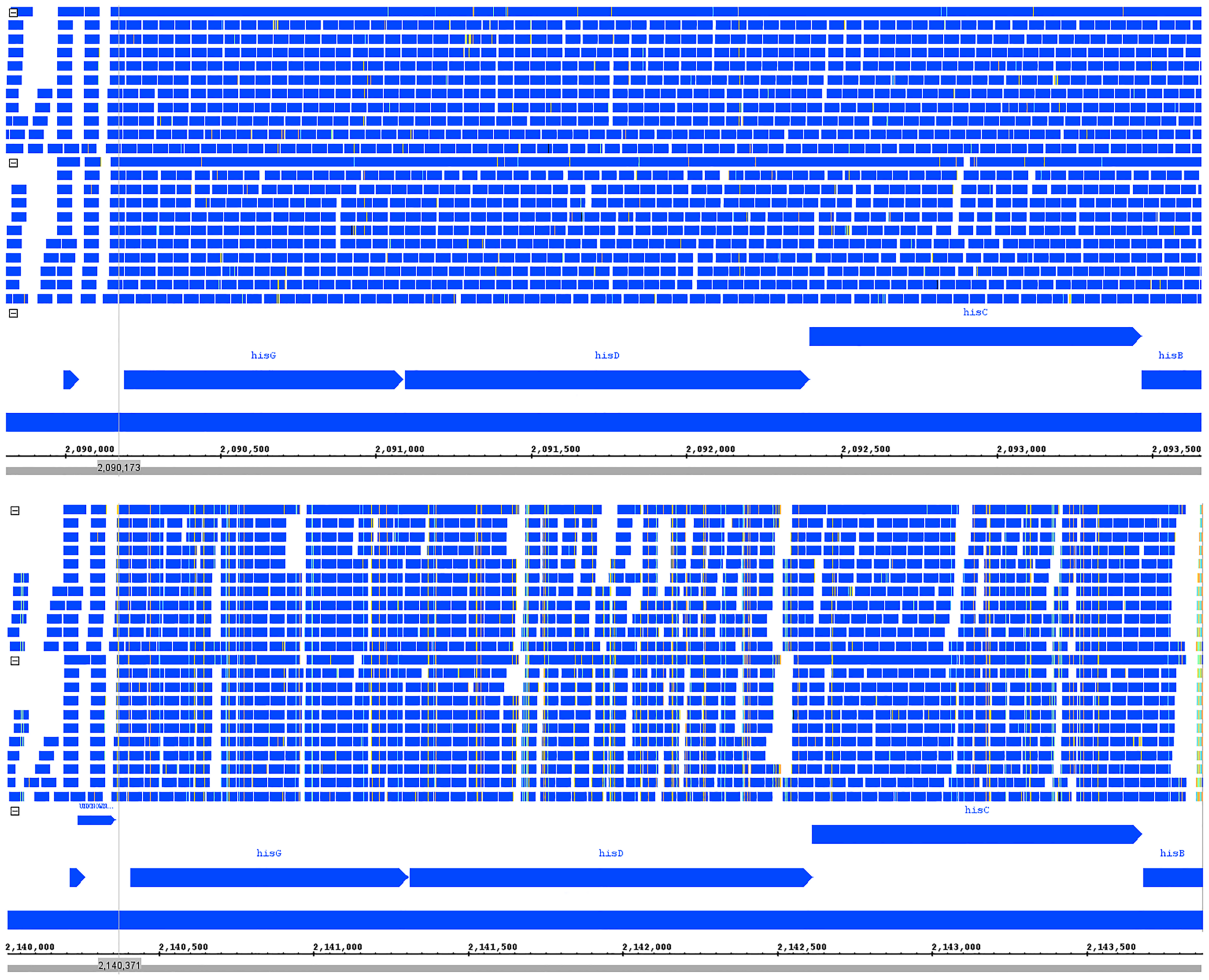
hisD reads aligned to native genome (top) and heterologous genome (bottom). Substantial read loss due to high-density SNP clusters can be seen in the non-native reference.

As a representative gene for false positives due to read alignment loss by SNPs, hisD, a gene which codes for histidinol dehydrogenase, was chosen. This gene was selected because it showed very uniform coverage in the native reference genome and because SNPs were distributed widely in various concentrations across the length of the heterologous reference, which makes read loss more visually apparent. Fig 7 shows read alignment for hisD from the *E*.*coli* batch condition, with the K12 strain being the native reference and the IAI1 strain being the heterologous reference. This gene has 55 SNPs between the native and heterologous genome across a length of 1305 bases. Read loss can be observed particularly in regions of high SNP density, where read alignment becomes increasingly more difficult due to differences in the reference sequences. The two flanking genes, hisG and hisC also show some moderate read loss, but these genes are not identified as false positives because the read loss is less severe and doesn’t cause a significant enough log-fold change to trigger differential expression flagging. Other genes identified as false positives show similar read loss when windows of high-density SNPs are present, with some cases having very distinct windows of loss and otherwise similar coverage between genomes, and still others showing staggered read loss across the gene, as was shown here in the case of hisD.

The type of read loss observed between native and heterologous genomes due to SNPs might be reduced by either relaxing read alignment parameters so that reads can be aligned when higher levels of SNPs are present, or otherwise by through the application of sequencing technologies that produce longer reads. One potential problem of approaching this issue by adjusting alignment parameters is that reads may incorrectly map with higher propensity to incorrect regions, further biasing the read set. This problem would be further compounded if only uniquely mapped reads were used, as the proportion of reads that map to multiple locations would necessarily increase as alignment parameters become less restrictive.

### Simulation of mapping at different read lengths

A majority of the false positives identified were caused by read loss in regions with high levels of SNPs. In order to examine if this effect can be mitigated by read length, several simulations were performed.

**Fig 8:**
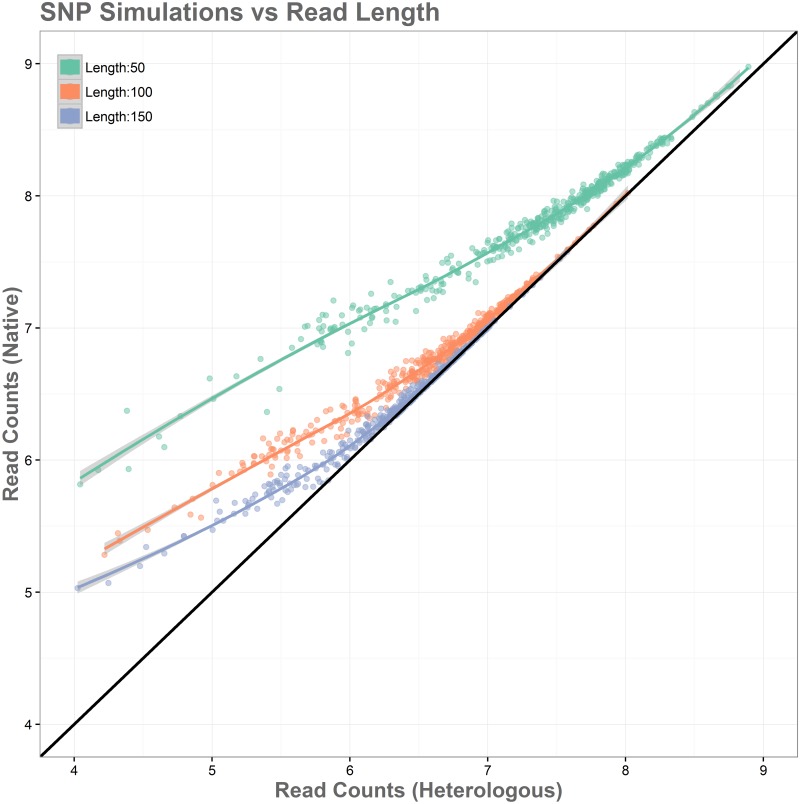
Simulation of the relationship between SNPs and read length with each gene containing 35 randomly distributed SNPs and simulated reads of length 50, 100, and 150. Log-fold changes in read alignment for native and heterologous genomes show that shorter reads perform poorly when aligned to heterologous genomes, while longer reads are more resilient.

**Fig 9:**
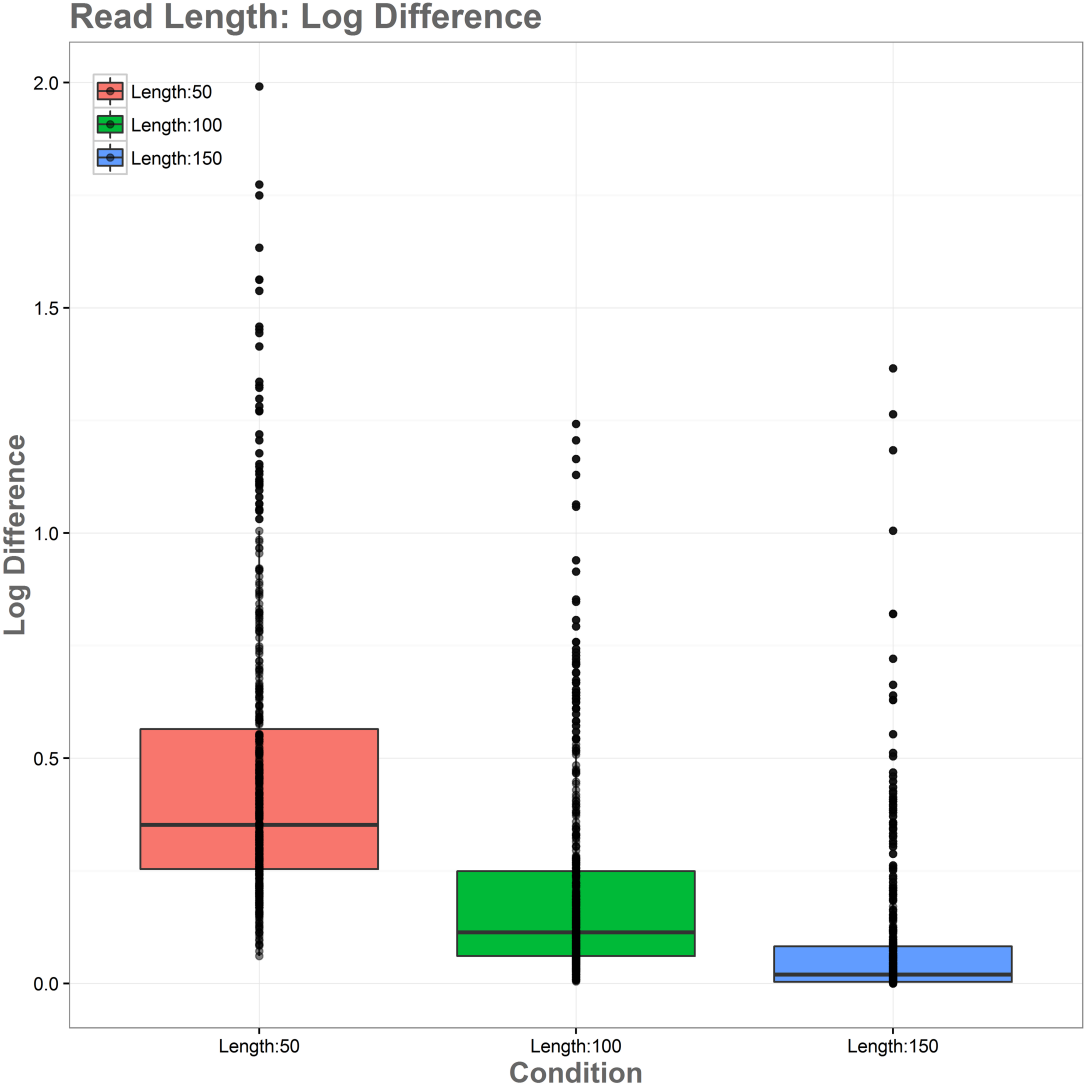
Log-fold differences in native vs heterologous alignment for different read lengths. Shorter reads handle mapping more poorly and are subject to significant bias in non-native alignments, while bias is minimalized with increasing read length.

The simulations showed substantial improvement in accurate alignments between native and heterologous reference genomes as read length increases. This relationship can be seen in Fig 8. Reads of length 50 performed the most poorly in simulations, with all genes showing read loss when aligned to the heterologous reference genome, and those with lower expression levels showing the greatest log-fold changes. Reads aligned to the heterologous genome with length 50 had 19.77% read loss overall. Reads of length 100 performed significantly better, with log-fold changes in read alignment being substantially closer to expected values and becoming increasingly reliable as expression levels increase. These reads showed a significantly better alignment, with a read loss of 8.33%. Reads of 150 in length showed the best performance among simulations, with higher accuracy for all reads over the entire range of expression levels and complete accuracy being reached at lower expression levels than the 50 and 100 read length simulations. Alignment here was again the best of the simulations, with a read loss of only 3.09%. Fig 9 shows an overview of log-fold differences between alignment to native and heterologous genomes for the three simulated read lengths. Overall these simulations show that heterologous reference use is more reliable with longer read lengths, and that the expected number of false positives caused by polymorphisms will be reduced as read length increases.

### Robustness of analysis with varying alignment sensitivity and read depth parameters

**Fig 10:**
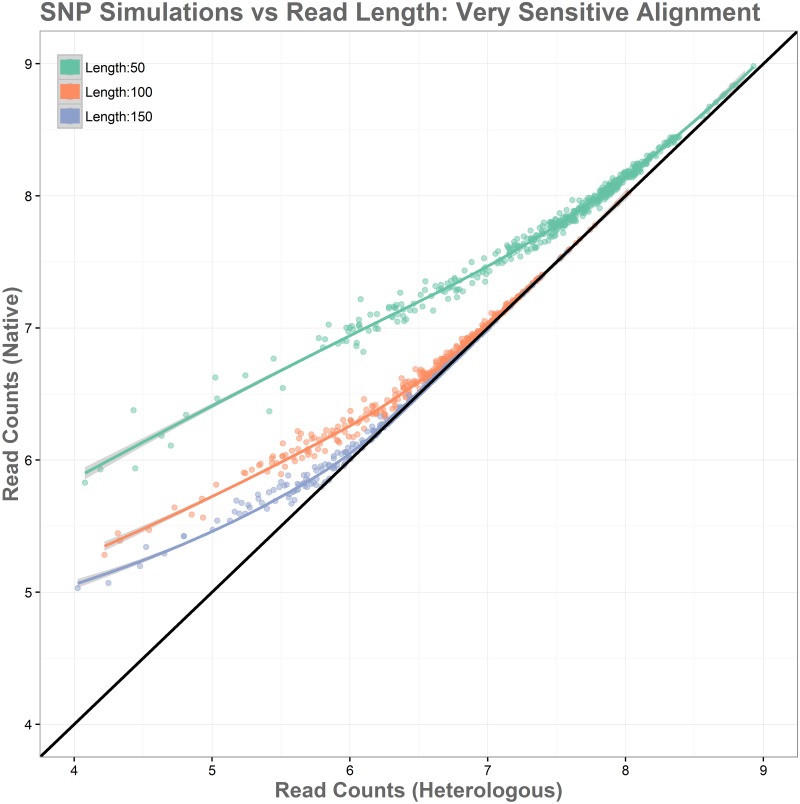
Simulation of the relationship between SNPs and read length shown using bowtie2’s—Very-sensitive alignment settings. Modest reduction in bias was observed in these simulations with regard to standard alignment parameters.

**Fig 11:**
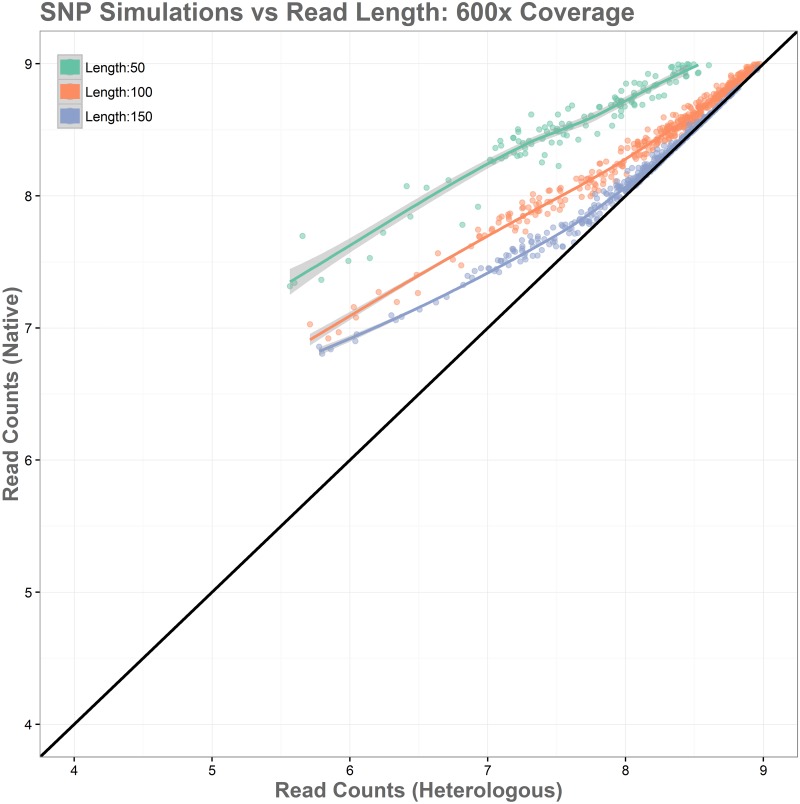
Simulation of the relationship between SNPs and read length shown at 600x coverage. Increased depth has little effect on bias, as relative read loss remains consistent in non-native alignments.

An additional variation of this simulation was performed using bowtie2’s “—very-sensitive” alignment parameter. The use of this argument increased overall alignment in all cases, reducing read loss to 13.65% for the 50 read length simulation, 4.00% for the 100 read length simulation, and to just 1.41% for the 150 read length simulation. This is a modest improvement over the standard alignment parameters and can be seen in Fig 10 as each curve becoming slightly tighter and approaching accurate read levels across native and heterologous genomes from slightly lower expression levels. The lower range of expression values, however, are not influenced strongly enough for this method to mitigate false positives completely, while it does have value and should be used for native and heterologous alignment issues, the stronger influence appears to come from increases in read length. Next, the effect of read depth was examined. In our sample data, *E*. *coli* had an average read coverage of 150x, *V*. *vulnificus* had a much greater read depth of around 600x. Simulations of these conditions show that increasing read depth has little influence, simply compressing the range of depth toward the higher end, and maintaining similar ratios of log-fold differences between native and heterologous genomes. The results of this simulation can be seen in Fig 11.

### Treatment of multiple and unique mapping positions

Some false positives were caused between native and heterologous genomes when insertion events that copied short segments into or adjacent to coding regions were present, causing reads from other regions of the genome to incorrectly map to some genes in the non-native genome. One possible approach to removing these false positives is to consider only reads that uniquely map to a single position in the genome as valid reads. This would mean that reads that map incorrectly would not be included in read counts, but also that those reads would not correctly map to their proper location as well. If the correct mapping location for these reads, however, was orthologous between the native and heterologous genomes, the bias introduced from removing these reads should be roughly the same, with the effect of eliminating false positives while maintaining a true ratio of gene expression for genes containing the correct mapping position.

To simulate the effects of this approach, Simulome was used to create a 500-gene simulated reference genome and a mutated variant with insertion events 100 bases in length, which were copied from other random positions in the original reference. This means that in each gene in the variant genome, an insertion of 100 base pairs exists that also has a correct mapping location elsewhere in the genome. Read data was created using ART for the simulated reference genome based on ART’s Illumina HiSeq 2500 model, with simulated fold coverage of 150.

**Fig 12:**
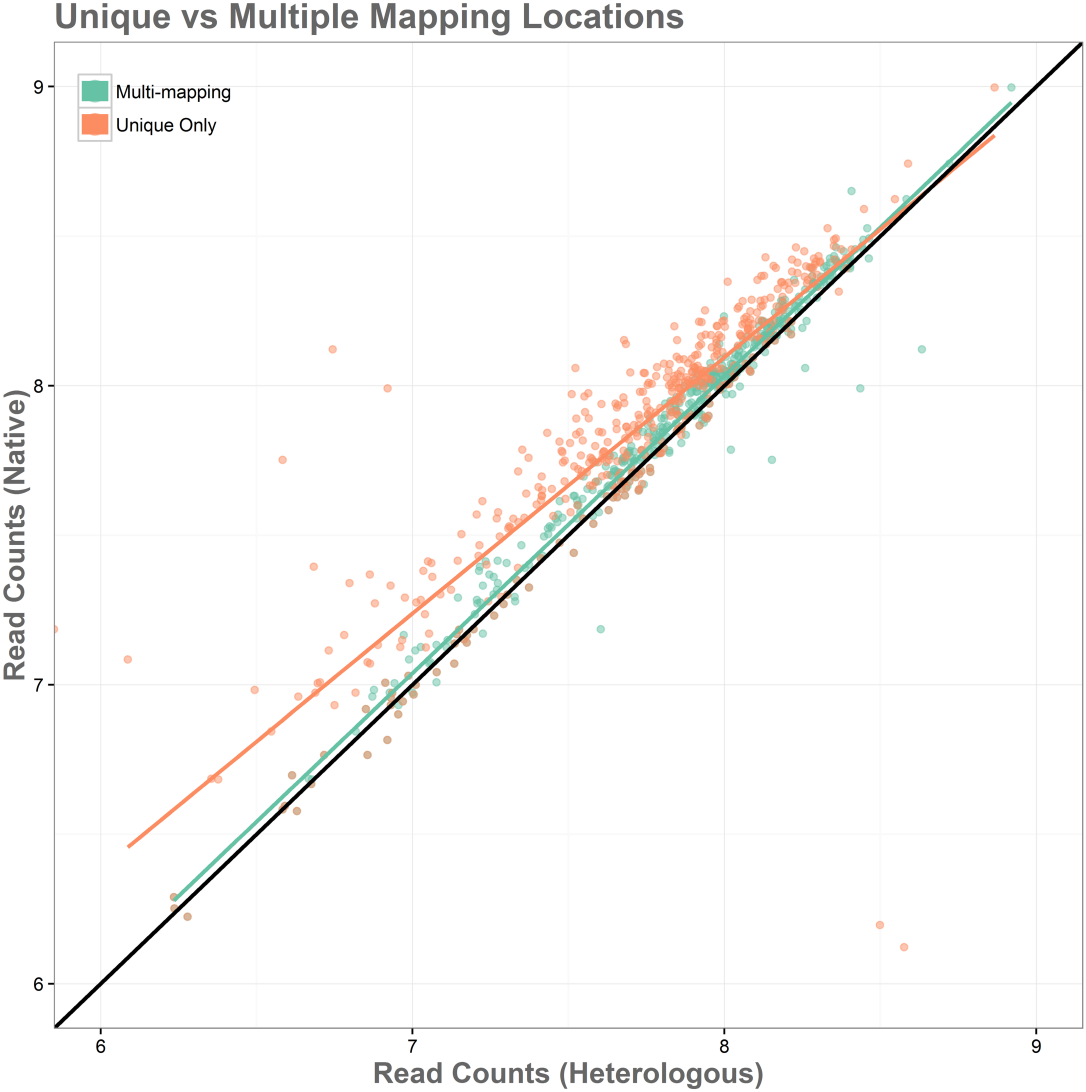
Simulation of the reads with ambiguously mapping inserts. Log-fold change in read alignment for native and heterologous genomes shows that less bias is present when Bowtie2 determines alignment for reads with ambiguous mapping positions.

Fig 12 shows the performance of read alignment for the native and heterologous genomes with ambiguously mapping positions included and only uniquely mapped positions. The condition in which multiple mapping locations were included performed much better, with most genes showing appropriate levels of read alignment across the native and heterologous genomes. This scenario did show several genes that would likely be identified as false positives, which can be seen as being more highly expressed in the heterologous genome. These genes were not present as false positives in the case of unique mapping and returned to a more appropriate read alignment ratio between the native and heterologous genomes, but overall the level of read alignment for the heterologous genome is reduced significantly overall, introducing a bias that is far more extreme than the problem it solves.

## Discussion

The use of non-native references genomes relies on the distance between the native and heterologous genomes and the development of high-integrity data that can overcome the naturally occurring differences between those genomes. Several factors should be taken into account by researchers intending to use non-native references for alignment of read sets. The first step that must always be taken is to identify correspondence between orthologous regions. For example, a researcher with a read set with no complete native reference genome available that has a potential heterologous genome available for read alignment should first investigate if the heterologous genome is viable for alignment. To do this, de novo assembly of reads into contigs, followed by ortholog identification using BLAST should be performed. Then, by extracting read counts for orthologous regions, correspondence can be examined as shown in Fig 3. It is important to mention that the parameters of ortholog identification here are highly relevant. In this study, we performed ortholog identification using very strict parameters (95% identity, length > 200bp, max mismatch = 5) and were able to identify a large subset of orthologous regions with high confidence. By relaxing these ortholog identification criteria, undoubtedly a larger subset of orthologous genes could be identified, however the false positive rate would also correspondingly increase with increased numbers of mismatching regions existing. A researcher intent on using a non-native reference genome for alignment should then properly tune their BLAST ortholog identification parameters to maximize the number of orthologs they can identify between their read set and non-native reference genome, while confirming viability by monitoring the correspondence of a single read set as aligned to both the non-native reference and their de novo assembled contig sets. That is, as long as an identical read set produces strong correspondence when aligned to the native and non-native alignment target, such as that seen in Fig 3 of this study, comparison between those orthologous regions can be considered viable. If that alignment instead becomes increasingly dispersed, the strictness of BLAST parameters for ortholog mapping should be increased. Once the researcher has determined an appropriate level of ortholog identification, additional investigation can be performed, if desired, to further eliminate false positive outliers.

In this study, we have observed that genomes with short reads are particularly vulnerable to false positives when using a heterologous genome for read alignment, even with very strict correspondence between orthologous regions. Most false positives originate from sites with a high frequency of polymorphic sites, with a few false positives being caused by other mutation events. Our *E*. *coli* samples, which used 50 base reads, contained several false positives that we were able to identify and subsequently analyze to gain insight into underlying causes of incorrect information that must be considered when working with non-native reference genomes. By contrast, our *V*. *vulnificus* data set showed that by using longer reads with more depth that false positives can be largely avoided, having almost no false positives at all between native and heterologous genomes. With this being the case, researchers using non-native reference genomes should be aware of these issues and take appropriate precautions in their analyses by confirming both proper identification of orthologous regions and the use of longer reads to mitigate incorrect alignments that result in false positives for heterologous alignment.

Additional accuracy can be achieved when necessary by researchers using heterologous reference genomes. By performing BLAST analysis between read sets and the reference genome to be used, potential false positives can be identified by searching for those reads which align with the highest ratio of polymorphic positions. While increasing read length is certainly the best way to avoid false positives and incorrect information when using a heterologous genome for read alignment, it is likely that as the distance between read data and reference genome increases, that the improvement observed through the use of longer reads would degrade. In general, if it is known that a heterologous genome will be used in a study, longer reads should be generated whenever possible. Additional improvements can be made by adjusting read alignment parameters, but these tend to be fairly modest, with false positives still being likely to occur even with strict alignment parameters. Overall the level of false positives in both cases is low, with the false positive discovery rate compounding as more complex comparisons involving more alignments of data are performed. Using a non-native reference genome for research seems to be a safe endeavor in general if genetic distances between native and non-native conditions are not excessively large, however for very sensitive experiments the use of heterologous reference genomes should be approached with caution, as it is possible that some important genes may be subject to bias without the benefit of a native reference genome.

In this study, we have examined the problems that can arise from the use of non-native, heterologous genomes as references for RNA-Seq read alignment. We have described a method for identifying false positives, outlined the underlying causes, and suggested a set of best practices for studies that use non-native reference genomes, that will allow researchers to make informed decisions about how they handle their data analyses. The analysis workflows described in this study can potentially be applied to novel data sets to help investigators estimate whether it is a safe assumption to use a common reference genome—either for ease of analysis, or because complete reference genomes for all species or strains in the study are not yet available. In the case where partial genomic information is available, reciprocal mapping analysis can be applied to orthologous genes in the unambiguously alignable portions. These regions can be analyzed to determine the level of correspondence of results between alternate mappings, and to identify the fraction of potential false positives in the analyzable subset of the data. While this will not provide a complete reciprocal analysis, it does provide a quantitative basis by which to justify use of a heterologous common reference for multiple strains, or potentially to justify the expense of finishing additional strain genomes to provide a more accurate reference if available genomes are not sufficient.
